# Presence of a Haloarchaeal Halorhodopsin-Like Cl^−^ Pump in Marine Bacteria

**DOI:** 10.1264/jsme2.ME17197

**Published:** 2018-03-29

**Authors:** Yu Nakajima, Takashi Tsukamoto, Yohei Kumagai, Yoshitoshi Ogura, Tetsuya Hayashi, Jaeho Song, Takashi Kikukawa, Makoto Demura, Kazuhiro Kogure, Yuki Sudo, Susumu Yoshizawa

**Affiliations:** 1 Atmosphere and Ocean research Institute (AORI), The University of Tokyo 5–1–5, Kashiwanoha, Kashiwa, Chiba 277–8564 Japan; 2 Department of Natural Environmental Studies, Graduate School of Frontier Sciences, the University of Tokyo Chiba 277–8563 Japan; 3 Graduate School of Medicine, Dentistry and Pharmaceutical Sciences, Okayama University Okayama 700–8530 Japan; 4 Department of Bacteriology, Faculty of Medical Sciences, Kyushu University Fukuoka-shi, Fukuoka 812–8582 Japan; 5 Department of Biological Sciences, Inha University Incheon 22212 Republic of Korea; 6 Faculty of Advanced Life Science, Hokkaido University Sapporo 060–0810 Japan; 7 Global Station for Soft Matter, Global Institution for Collaborative Research and Education, Hokkaido University Sapporo 060–0810 Japan

**Keywords:** family *Rhodothermaceae*, rhodopsin, genome analysis, chloride ion pump

## Abstract

Light-driven ion-pumping rhodopsins are widely distributed among bacteria, archaea, and eukaryotes in the euphotic zone of the aquatic environment. H^+^-pumping rhodopsin (proteorhodopsin: PR), Na^+^-pumping rhodopsin (NaR), and Cl^−^-pumping rhodopsin (ClR) have been found in marine bacteria, which suggests that these genes evolved independently in the ocean. Putative microbial rhodopsin genes were identified in the genome sequences of marine *Cytophagia*. In the present study, one of these genes was heterologously expressed in *Escherichia coli* cells and the rhodopsin protein named *Rubricoccus marinus* halorhodopsin (RmHR) was identified as a light-driven inward Cl^−^ pump. Spectroscopic assays showed that the estimated dissociation constant (*K*_d,int._) of this rhodopsin was similar to that of haloarchaeal halorhodopsin (HR), while the Cl^−^-transporting photoreaction mechanism of this rhodopsin was similar to that of HR, but different to that of the already-known marine bacterial ClR. This amino acid sequence similarity also suggested that this rhodopsin is similar to haloarchaeal HR and cyanobacterial HRs (*e.g.*, SyHR and MrHR). Additionally, a phylogenetic analysis revealed that retinal biosynthesis pathway genes (*blh* and *crtY*) belong to a phylogenetic lineage of haloarchaea, indicating that these marine *Cytophagia* acquired rhodopsin-related genes from haloarchaea by lateral gene transfer. Based on these results, we concluded that inward Cl^−^-pumping rhodopsin is present in genera of the class *Cytophagia* and may have the same evolutionary origins as haloarchaeal HR.

Rhodopsins are seven-transmembrane proteins composed of a protein moiety (opsin) and light-absorbing chromophore (retinal). They are widely distributed among animals and prokaryotes and have been classified into two groups: type-1 (microbial origin) and type-2 (eukaryotic origin). Microbial type-1 rhodopsins were initially discovered in extremely halophilic archaea: a light-driven outward H^+^-pumping rhodopsin (bacteriorhodopsin [BR] [26]), inward Cl^−^-pumping rhodopsin (halorhodopsin [HR] [22]), and light-sensing rhodopsins (sensory rhodopsin I and II [SRI and SRII]) (31). In 2000, an H^+^-pumping rhodopsin was also found in a marine bacterial genomic fragment using a metagenomic approach and was named proteorhodopsin (PR) (4). Recent studies have shown that PR genes are widely present among marine bacteria and archaea inhabiting the euphotic layer of the ocean (7). Furthermore, outward Na^+^-pumping rhodopsin (NaR) (15) and inward Cl^−^-pumping rhodopsin (ClR) (43) were discovered in marine bacteria. Of note, BR and HR of haloarchaea and PR and ClR of marine bacteria belong to distinct phylogenetic lineages, suggesting that these ion-pumping rhodopsins evolved independently in their respective habitats.

The family *Rhodothermaceae* belongs to the class *Cytophagia* in the phylum *Bacteroidetes*. This family includes 7 genera (*Longimonas* [40], *Longibacter* [41], *Rhodothermus* [1], *Rubricoccus* [27], *Rubrivirga* [28], *Salinibacter* [3], and *Salisaeta* [36]) and has been found in diverse environments, *e.g.*, hydrothermal vents, hypersaline environments, and oceans. The genera *Longimonas*, *Longibacter*, *Salisaeta*, and *Salinibacter* include halophilic bacteria, and members of the genus *Rhodothermus* are thermophiles. In contrast, members of the genera *Rubricoccus* and *Rubrivirga* have been isolated from surface or deep seawater and are not extremophiles. *Salinibacter*, a halophilic bacterium, dominates with halophilic archaea in hypersaline environments (5–25% according to fluorescence *in situ* hybridization [2] and 7–9% according to a metagenomic analysis [8]). Previous studies reported that *Salinibacter ruber* M31^T^ carries multiple rhodopsin genes: xanthorhodopsin (XR), HR, and SRI (23). In addition, a genomic analysis suggested that *S. ruber* acquired not only the HR and SRI genes, but also many other genes that are necessary for salt adaptation from halophilic archaea by lateral gene transfer (LGT) because both of these halophilic prokaryotes inhabit and dominate the same environment (23).

Following the genome information of *Rubricoccus marinus* SG-29^T^ (24), we herein report two genome sequences of *Rubrivirga marina* SAORIC-28^T^ and *R. profundi* SAORIC-476^T^. A genomic analysis revealed that *R. marinus* SG-29^T^ and *R. marina* SAORIC-28^T^ have five rhodopsin genes belonging to two different clusters: one is included in the xenorhodopsin (XeR) cluster, and the other is a unique cluster consisting of rhodopsin containing a TSA motif (amino acid residues 85, 89, and 96 in BR numbering). A phylogenetic analysis showed that this unique rhodopsin cluster is closely related to the HR of halophilic archaea. Therefore, we named these HR-like rhodopsins RmHR (*R. marinus* SG-29^T^ halorhodopsin), R28HR1, and R28HR2 (*R. marina* SAORIC-28^T^ halorhodopsin 1 and 2). A functional analysis using heterologously expressed RmHR was subsequently conducted to identify its ion specificity. We further performed a spectroscopic analysis using purified RmHR to clarify its spectroscopic characteristics because even rhodopsins with the same ion transport specificity have intermediates with different ion affinities and structural changes, and the properties are important factors that define rhodopsins. In the present study, we performed genomic and phylogenetic analyses, and also characterized RmHR spectroscopically, and these properties were compared with those of known microbial rhodopsins.

## Materials and Methods

### Strain information and genomic sequencing of *R. marinus* SG-29^T^,* R. marina* SAORIC-28^T^, and *R. profundi* SAORIC-476^T^

*R. marinus* SG-29^T^ was isolated from surface seawater (30° 40′ N, 138° 00′ E; depth 50 m), and additional information has been reported previously (27). *R. marina* SAORIC-28^T^ was isolated from a deep seawater sample obtained from the western North Pacific Ocean (32° 00′ N, 138° 13′ E; depth 3000 m) by the R/V *Tansei maru* (Atmosphere and Ocean Research Institute, The University of Tokyo and Japan Agency for Marine-Earth Science and Technology [JAMSTEC]) on 3 July 2010 (KT-10-12 cruise). *R. profundi* SAORIC-476^T^ was isolated from a deep seawater sample obtained from the western North Pacific Ocean (32° 00′ N, 140° 00′ E; depth 3000 m) during the research cruise (MR-11-05) of R/V *Mirai* (JAMSTEC) in May 2011. Details of the culture conditions and phenotypic traits of these strains have been described previously (28, 30). The genomic DNA of strains SAORIC-28^T^ and SAORIC-476^T^ was extracted using phenol-chloroform and ethanol precipitation (21). Library preparation was performed for strain SAORIC-28^T^, as previously reported for strain SG-29^T^ (24). A KAPA HyperPlus Kit (Kapa Biosystems, Boston, MA, USA) was used for library preparation for strain SAORIC-476^T^. Each end of the libraries (300 bp) of SAORIC-28^T^ and SAORIC-476^T^ were sequenced on a MiSeq instrument with MiSeq Reagent kit version 3 (Illumina, San Diego, CA, USA).

### Gene preparation and ion transport measurements of RmHR

The DNA fragments encoding RmHR, R28HR1, and R28HR2 were chemically synthesized by Eurofins Genomics (Eurofins Genomics, Tokyo, Japan) with codon optimization for *Escherichia coli*. These gene fragments were inserted into the NdeI and XhoI sites of the pET21a vectors (Novagen, Darmstadt, Germany); consequently, the plasmids encoded RmHR, R28HR1, and R28HR2 with hexahistidines at the C terminus. The vectors were transformed into *E. coli* strain C41 (DE3) (Lucigen, Middleton, WI, USA). *E. coli* cells with the plasmid were incubated at 37°C on an LB medium agar plate supplemented with ampicillin (final concentration, 100 μg mL^−1^). Cells carrying the plasmid were then grown at 37°C in 200 mL of 2×YT medium supplemented with ampicillin (final concentration, 100 μg mL^−1^), and protein expression was induced at an OD 660 nm (OD_660_) of 0.4–0.6 with 0.2 mM isopropyl β-D-1-thiogalactopyranoside (IPTG) and 10 μM all-*trans*-retinal (Sigma-Aldrich, St Louis, MO, USA). Rhodopsin-expressing cells were collected by centrifugation (8,000×*g* for 3 min), washed three times with 100 mM NaCl, and then re-suspended in the solvent for measurement. The light source was a 300 W xenon lamp (MAX-303; Asahi Spectra, Tokyo, Japan), and 6 mL of the cell suspension was initially placed in the dark and then irradiated through a 520±10 nm bandpass filter (MX0520; Asahi Spectra) for 3 min. pH was measured using a pH meter (LAQUA F-72; HORIBA, Kyoto, Japan). Measurements were repeated under the same conditions after the addition of the protonophore carbonyl cyanide m-chlorophenylhydrazone (CCCP, final concentration, 30 μM). All measurements were performed at 4°C. Different solutes (100 mM NaCl, KCl, MgCl_2_, NaBr, NaI, NaNO_3_, and Na_2_SO_4_) were used in the ion selectivity analysis of RmHR.

### Spectroscopic analysis of RmHR

*E. coli* BL21 (DE3) cells were used for the functional expression of RmHR as a recombinant protein. The procedures for protein expression and purification were the same as those described in our previous study (35). In spectroscopic measurements, the buffer was sufficiently exchanged by centrifugation (AmiconUltra centrifuge unit; 30,000 molecular weight cut-off, Merck Millipore, Bedford, MA, USA) and the PD-10 column (GE Healthcare, Waukesha, WI, USA) with 10 mM 3-(*N*-morpholino)-propanesulfonic acid (MOPS) buffer (Dojindo Laboratories, Kumamoto, Japan) containing 0.1% (w/v) *n*-dodecyl-β-D-maltoside (DDM, Dojindo Laboratories) and NaCl at the desired concentrations (1 mM to 4 M). In order to maintain ionic strength at 4 M, Na_2_SO_4_ was added to the samples at the desired concentrations (0–1.333 M). Neither Na_+_ nor SO_4_^2−^ was a substrate ion for RmHR (see the Results and Discussion section). The optical density of DDM-solubilized RmHR was adjusted to approximately 0.5 optical density (OD) at its absorption maximum (approx. 10 μM).

Retinal isomer compositions were analyzed using normal-phase high-performance liquid chromatography (HPLC). The procedures for the retinal oxime extraction and HPLC analysis were the same as those described in our previous study (35). Regarding measurements under dark conditions, the sample was maintained in the dark for more than 1 week. In measurements under light conditions, the sample was illuminated with 540±10 nm light for 5 min before retinal oxime extraction. The retinal oxime extracted from the purple membrane (PM) containing *Halobacterium salinarum* bacteriorhodopsin (HsBR) was used as a reference. The eluting retinal oxime was monitored by its absorption at 360 nm. The flow rate was 1 mL/min. All measurements were performed at 25°C.

All UV-Vis spectra were recorded at 25°C using a UV-1800 spectrophotometer (Shimadzu, Kyoto, Japan). In the Cl^−^ titration experiment, the absorption maximum wavelength (*λ*_max_) of RmHR was plotted against the logarithm of the Cl^−^ concentration ([Cl^−^]) and analyzed by fitting the data to the following Hill equation:

$$\lambda_{max}=a+b\cdot {\left[Cl^{-}\right]}^{n}/({\left[Cl^{-}\right]}^{n}+{\left[K_{d}\right]}^{n})$$

where *a*, *b*, *K*_d_, and *n* represent the offset, the amplitude of the *λ*_max_ change, the dissociation constant for Cl^−^, and the Hill coefficient, respectively. In the pH titration experiment, the sample was suspended in a 6-mix buffer (0.89 mM citrate, 0.89 mM MES, 1.1 mM TES, 0.78 mM TAPS, 1.1 mM CHES, 0.33 mM CAPS; MES, TES, TAPS, CHES, and CAPS are Good’s buffers and were purchased from Dojindo Laboratories) (34) containing 0.05% DDM, 0–4 M NaCl, and 0–1.333 M Na_2_SO_4_. The initial pH was approximately 3.8 and was then adjusted to the desired value by adding a very small amount of 2 M NaOH, followed by measurements of the UV-Vis absorption spectrum. The concentration change in the RmHR sample was almost negligible. The acid dissociation constant, p*K*_a_, of the protonated Schiff base of the retinal chromophore was estimated by fitting the data to the Henderson-Hasselbalch equation:

$$\Delta A=1/(1+10^{\left(pKa-pH\right)})$$

where *pH* and Δ*A* are the adjusted pH value and absorption change at 380 nm, which corresponds to the deprotonated Schiff base, respectively.

Time-dependent changes in absorbance were measured by flash-photolysis using a homemade computer-controlled apparatus equipped with a Nd:YAG laser (5 mJ pulse^−1^, 532 nm, 7 ns) as an actinic light source as described previously (18, 35). Due to the setting for the response time (0.5 μs) of a homemade *I*-*V* converter, a large scattering artifact from the laser flash appeared before 10 μs. Therefore, plots were started from 10 μs. Time-dependent changes in absorbance were measured from 400 to 710 nm at 10-nm intervals. Data before the laser pulse were adopted as a baseline. In order to improve the signal-to-noise ratio, the results of 30 flashes were averaged at each wavelength. No further data processing was applied. The temperature was kept at 20°C using a thermostat.

We analyzed the data using a sequential model based on our previous studies (10, 18) as follows:

$$P_{0}\to P_{1}\to P_{2}\to P_{3}\to P_{4}\to P_{0}$$

where P_0_ and P_1_–P_4_ represent the unphotolyzed original pigment and the 1^st^–4^th^ kinetically defined states, respectively. All data on time-dependent changes in absorbance were simultaneously fit with a sum of 4 exponential decay functions. The appropriate number of exponents was selected based on reductions in the standard deviation of the residuals. The P_1_–P_4_ states were allowed to contain a few physically defined photointermediates such as L, N, and O with individual absorption maxima when a quasi-equilibrium state existed between them. The time constants *τ*_1_–*τ*_4_ and the absorbance differences Δ*ɛ*_1_–Δ*ɛ*_4_ between the P_1_–P_4_ states and the original *P*_0_ state were assessed according to the fitting results. Independently, the pure retinal spectrum of the P_0_ state was extracted from the UV-Vis absorption spectrum of the initial state (unphotolyzed) RmHR by spectral decomposition using the skewed Gaussian function as described previously (10). Finally, the absolute spectra of each P_1_–P_4_ state were obtained by adding the P_0_ spectrum to each absorbance difference Δ*ɛ*_1_–Δ*ɛ*_4_.

As described in the Results and Discussion section, the P_3_ state was obtained as a quasi-equilibrium mixture of N-like and O-like intermediates with relative fractions that were dependent on the Cl^−^ concentration. P_3_ state spectra contained not only the main absorption bands of the N-like and O-like intermediates, but also the respective β-bands. The procedures for the spectral decomposition of the P_3_ state were the same as those described in previous studies (10, 29). Assuming both β-bands are identical to that of the P_0_ state, the spectrum of the P_3_ state is described as:

$$Spectrum\;of\;the\;P_{3}\;state=f\times \text{Abs}(N,\lambda )+(1-f)\times \text{Abs}(O,\lambda )+\text{Abs}(\beta ,\lambda )$$

where *f*, Abs (*N, λ*), Abs (*O, λ*), and Abs (*β, λ*) represent the fraction of the N-like intermediate, the main absorption band of the N-like intermediate, the main absorption band of the O-like intermediate, and the β-band, respectively. We employed skewed Gaussian functions to describe these three absorption bands and then decomposed the spectrum of the P_3_ state to estimate *f*, which we assumed to be the only parameter dependent on the Cl^−^ concentration. The fractions of the N-like and O-like intermediates were plotted against the Cl^−^ concentration, and data were simultaneously analyzed by the Hill equation as follows:

$$Fraction\;of\;N-like\;(or\;O-like)=a+b\cdot {\left[Cl^{-}\right]}^{n}/({\left[Cl^{-}\right]}^{n}+{\left[K_{d}\right]}^{n})$$

where *a*, *b*, *K*_d_, and *n* represent the offset, amplitude of the *λ*_max_ change, dissociation constant for Cl^−^, and Hill coefficient, respectively.

### Phylogenetic and comparative genome analyses

Multiple alignments of amino acid sequences were performed using the CLUSTALW option in MEGA 6.0 software (32). Phylogenetic trees were constructed using the maximum-likelihood (ML) method (5) with 1,000 bootstrap replications (6). The nucleotide sequences of 16S rRNA and the amino acid sequences of rhodopsins, 15,15′-β-carotene dioxygenase (*blh*), and lycopene cyclase (*crtY* and *crtYcd*) genes were collected from the NCBI database and RAST annotated sequences for the genera *Rubricoccus* and *Rubrivirga*. In order to compare gene synteny around the Cl^−^-pumping rhodopsin gene and *blh* gene, we acquired the genomes of *H. salinarum* R1, *S. ruber* M31^T^, *Synechocystis* sp. PCC 7506, *Mastigocladopsis repens* PCC 10914, *Salisaeta longa* DSM 21114^T^, and *Rhodothermus marinus* DSM 4252^T^ from the NCBI RefSeq database. The ortholog clustering of the CDSs of each genome was performed using eggNOG-mapper (13) and the bactNOG dataset in the eggNOG database, version 4.5 (14). We visualized the operon structure and colored each gene based on each eggNOG ortholog cluster using R software. In order to evaluate the potential LGT, a BLAST analysis was performed on the genomes of *H. salinarum* R1, genes with an e-value less than 1e-10 were counted, and a threshold with a sequence identity >50% was established. We used the genome family *Rhodothermaceae*, *Cytophaga hutchinsonii* ATCC 33406^T^, which is a type species of the class *Cytophagia*, *Indibacter alkaliphilus* LW1^T^, *Spirosoma linguale* DSM 74^T^, *N. marinus* S1-08^T^, and *Flavobacterium aquatile* LMG 4008^T^. Since *I. alkaliphilus* and *S. linguale*, belonging to the class *Cytophagia*, have a rhodopsin gene, these genome data were used for this assay. Furthermore, because *N. marinus* is a marine flavobacterium with the ClR gene and *F. aquatile* is a type species of the class *Flavobacteriia*, these genome data were also used.

## Results and Discussion

### Genomic and phylogenetic analysis of *R. marinus* SG-29^T^,* R. marina* SAORIC-28^T^, and *R. profundi* SAORIC-476^T^

**Fig. 1:**
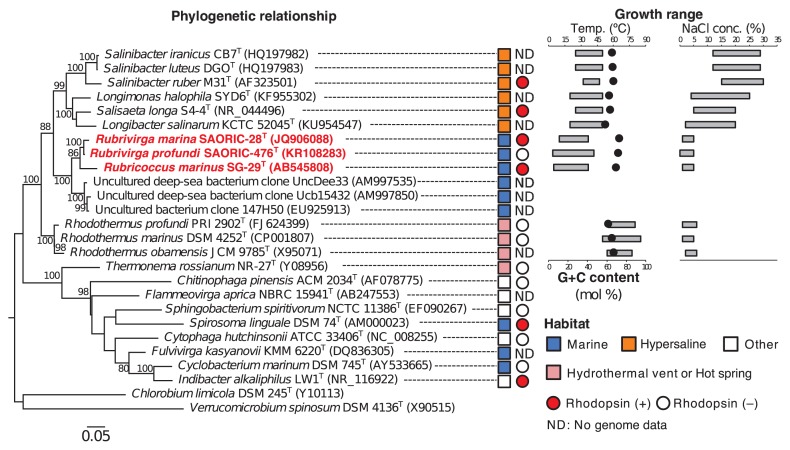
Maximum-likelihood phylogenetic tree based on 16S rRNA gene sequences. Phylogenetic tree of 16S rRNA showing the taxonomic positions of strains SG-29^T^, SAORIC-28^T^, and SAORIC-476^T^ among related species of the family *Rhodothermaceae* in the classes *Cytophagia* and *Sphingobacteria*. The habitats of each strain are indicated by different-colored squares. The red and open circles indicate strains containing the rhodopsin gene and no rhodopsin gene, respectively. The G+C content and growth range for temperature and NaCl concentrations are also shown on the right side. Black closed circles indicate G+C content of each strain. ND: No genome data.

A previous study showed that *R. marinus* SG-29^T^ has a 4.43-Mbp chromosome encoding 3847 CDSs (24). The present genome sequence analysis of *R. marina* SAORIC-28^T^ and *R. profundi* SAORIC-476^T^ revealed that these strains have 4.98 Mbp and 4.48 Mbp chromosomes encoding 4267 and 3842 CDSs, respectively (Table S1). The G+C contents of both strains from the genus *Rubrivirga* were two of the highest (72.5% and 71.3%) among the available genomes of class *Cytophagia* in the NCBI database. The genera *Rubrivirga* and *Rubricoccus* belong to the family *Rhodothermaceae*, and the 16S rRNA phylogenetic tree is shown in Fig. 1. This family contains members of thermophilic (genus *Rhodothermus*), halophilic (genera *Salinibacter*, *Salisaeta*, *Longimonas*, and *Longibacter*), and slightly halophilic (genera *Rubricoccus* and *Rubrivirga*, isolated from ocean) strains. Growth ranges for the NaCl concentration of thermophilic strains were similar to those of the genera *Rubricoccus* and *Rubrivirga* (Fig. 1). In contrast, halophilic bacteria in the family *Rhodothermaceae* grew under high-salinity conditions (20–30%). In addition, the lower limit of the growth range for NaCl of the genera *Longimonas*, *Longibacter*, and *Salisaeta* (2–4%), was lower than that of the genus *Salinibacter* (12–15%). The temperature growth range for the genera *Rubricoccus* and *Rubrivirga* was approximately 4–42°C (Fig. 1). In contrast, halophilic *Rhodothermaceae* strains grew at 20–50°C, and this range was between the temperature growth range for thermophiles (50–85°C) and marine isolates (4–42°C). These physiological traits clearly showed that the family *Rhodothermaceae* contained two different types of extremophiles, indicating that the common ancestor of this family was a halophilic or thermophilic bacterium. Therefore, the genera *Rubricoccus* and *Rubrivirga* with members that inhabit the ocean may have a higher G+C content in class *Cytophagia* as a remnant of their ancestors.

**Fig. 2:**
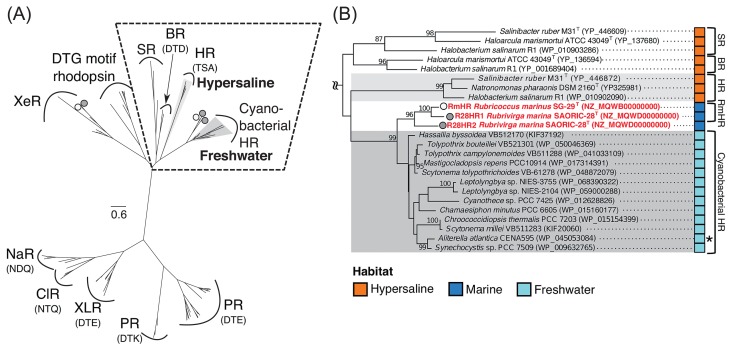
Unrooted maximum-likelihood phylogenetic tree of microbial rhodopsins. (A) Amino acid sequences of microbial rhodopsins were aligned using CLUSTALW, and evolutionary distances were estimated using the LG with Freg model. The tree was constructed using bootstrap values based on 1,000 replications; evolutionary analyses were conducted in MEGA 6.0. All rhodopsin amino acid sequence data used in this study were obtained from the public database (http://www.ncbi.nlm.nih.gov/). Dark gray indicates the clade consisting of the freshwater cyanobacterial rhodopsin clade, and light gray indicates halorhodopsin. An open circle and closed gray circle indicate the positions of strain SG-29^T^ and SAORIC-28^T^, respectively. Sodium ion pump rhodopsin (NaR); chloride ion pump rhodopsin (ClR); xanthorhodopsin-like rhodopsin (XLR); proteorhodopsin (PR); xenorhodopsin (XeR); sensory rhodopsin (SR); bacteriorhodopsin (BR); halorhodopsin (HR). The motif sequence (amino acid residues 85, 89, and 96 in BR numbering) is shown under the rhodopsin name. (B) Detailed phylogenetic tree of the SR, BR, HR, cyanobacterial HR, and RmHR homologues. The habitats of each strain are indicated by squares of different colors. Bootstrap values >80% are indicated as a percentage of the replicates tested. Asterisk represents the strain that was isolated from seawater by using medium for freshwater cyanobacteria.

The genome sequence analysis showed that the genomes of *R. marinus* SG-29^T^ and *R. marina* SAORIC-28^T^ encoded two different types of rhodopsin genes (RmXeR [WP_094549673] and RmHR [WP_094550238]) and three rhodopsin genes (R28XeR [WP_095509440], R28HR1 [WP_095509924], and R28HR2 [WP_095512583]), respectively. The identities of amino acid sequences among RmHR, R28HR1, and R28HR2 were 73% (RmHR-R28HR1), 62% (RmHR-R28HR2), and 57% (R28HR1-R28HR2) respectively. No rhodopsin gene was found in the genome of *R. profundi* SAORIC-476^T^. Although R28HR1 and R28HR2 were found from the deepsea isolate, it currently remains unclear whether these are deep-sea adapted rhodopsins because the habitat of the genus *Rubrivirga* is not yet understood. In the BLASTP search against the metagenome database (env_nr), no sequence with >50% identity with RmHR was found. This result implies that the RmHR gene is very rare in the marine environment. In the family *Rhodothermaceae*, four strains (*S. ruber* M31^T^, *S. longa* DSM 21114^T^, *R. marinus* SG-29^T^, and *R. marina* SAORIC-28^T^) have rhodopsin genes. In contrast, no rhodopsin gene was found in the two thermophilic strains belonging to the genus *Rhodothermus* (Fig. 1). A recent study showed that RmXeR of SG-29^T^ functions as a light-driven inward proton pump (17). R28XeR also belongs to the same cluster of RmXeR, suggesting that the function of R28XeR is an inward H^+^ pump. RmHR, R28HR1, and R28HR2 formed a unique cluster close to HR and SyHR (Fig. 2 and S1). Although HR and SyHR were discovered from halophilic prokaryotes and a freshwater cyanobacterium, RmHR, R28HR1, and R28HR2 formed a unique marine bacterial cluster. RmHR and R28HR1 had the same TSA motif as HR, and R28HR2 has TTD motif sequences (Table 1). In contrast, cyanobacterial HRs had the TSD, TSV, or TSL motif sequence (9).

### Light-induced pH changes and ion selectivity of RmHR

**Fig. 3:**
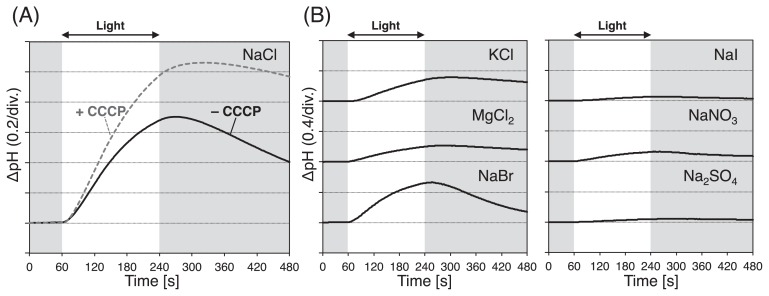
Anion transport activity of RmHR. Light-induced pH changes in *E. coli* cell suspensions expressing RmHR in solutions containing (A) 100 mM NaCl without (black solid line) and with (gray broken line) 30 μM CCCP and (B) 100 mM KCl, MgCl_2_, NaBr, NaI, NaNO_3_, and Na_2_SO_4_ without CCCP. The cell suspension was illuminated with green light (520 nm). Temperature was maintained at 4°C. The word “div.” represents division.

In order to clarify the function of this unique rhodopsin cluster, codon-optimized RmHR, R28HR1, and R28HR2 genes were chemically synthesized and each gene was heterologously overexpressed in *E. coli*. Light-induced pH changes were observed in suspensions of *E. coli* cells expressing each gene in 100 mM NaCl. However, light-induced pH changes in R28HR1 were smaller than those in RmHR, and were not observed in R28HR2. Therefore, RmHR was used in subsequent analyses. Light-induced alkalization was observed in 100 mM NaCl, and this pH change was not abolished by the addition of CCCP (Fig. 3A). Since CCCP is a protonophore, this alkalization may be explained by passive proton influx due to the negative membrane potential, which was created by outward Na^+^ or inward Cl^−^ translocation. In order to elucidate the ion selectivity of RmHR, we performed similar observations in different salt solutions (Fig. 3B). The signal almost disappeared in 100 mM NaI and Na_2_SO_4_, suggesting that RmHR cannot transport Na^+^. In contrast, strong signals were observed in 100 mM KCl, NaBr, and MgCl_2_, and a slight pH change was noted in 100 mM NaNO_3_. These anion-dependent transport properties are very similar to those of archaeal HRs in halophilic archaea, suggesting that RmHR functions as a light-driven inward anion (Cl^−^, Br^−^, and NO_3_^−^) pump.

### Spectroscopic analysis of RmHR

In order to investigate the spectroscopic properties of RmHR, we prepared a detergent DDM-solubilized sample. Under this condition, the results described below were able to be precisely compared to those of all the other anion-pumping rhodopsins investigated, such as *Natronomonas pharaonis* halorhodopsin (NpHR), *H. salinarum* halorhodopsin (HsHR), *Nonlabens marinus* S1-08^T^ rhodopsin 3 (NM-R3), *Synechocystis* sp. PCC 7509 halorhodopsin (SyHR), and *Mastigocladopsis repens* PCC 10914 halorhodopsin (MrHR) (11, 25, 33, 35, 42). Based on this background, we used DDM-solubilized RmHR in the subsequent spectroscopic analysis. We also focused on the photochemical properties of RmHR in the presence of NaCl because Cl^−^ is considered to be a candidate substrate anion for RmHR in the native habitat of *R. marinus* SG-29^T^ (Cl^−^ concentration of approximately 500 mM).

We initially examined the retinal isomer composition of RmHR under dark and light conditions in the presence of 1 M NaCl. As shown in Fig. S2A, RmHR predominantly possesses all-*trans* retinal as a chromophore, which is responsible for the light-driven anion pump function, at 99.6 and 96.2% under dark and light conditions, respectively. This property is the same as those of the other anion-pumping rhodopsins reported to date (11, 25, 33, 35). On the other hand, another rhodopsin encoded in the *R. marinus* SG-29^T^ gene (RmXeR) showed a light-dependent retinal composition change in the initial state (75 and 45% of all-*trans* retinal under dark and light conditions, respectively) (17). The biological significance of RmXeR currently remains unclear.

In order to evaluate this feature in RmHR, we measured the UV-Vis absorption spectra under various Cl^−^ concentrations ranging between 1 mM and 4 M (Fig. S2B). Many anionpumping rhodopsins show a spectral blue-shift upon substrate anion binding. In the experiment, ionic strength was maintained at 4 M by adding Na_2_SO_4_ at appropriate concentrations because in many Cl^−^-pumping rhodopsins, Cl^−^ binding reaches a plateau at 4 M and SO_4_^2−^ is not a substrate ion for RmHR, as shown in Fig. 3B. As shown in Fig. S2B, RmHR exhibited a Cl^−^-dependent spectral blue-shift from 550 nm in the presence of 1 mM Cl^−^ to 542 nm in the presence of 4 M Cl^−^. Based on this dependency, Cl^−^-binding affinity in the initial state (practically the dissociation constant, *K*_d,int._) was estimated to be 7.6±1.7 mM by the Hill equation (Fig. S2C). The Hill coefficient was 1.2±0.23, indicating positive cooperativity for Cl^−^ binding. The estimated *K*_d,int._ value of RmHR was in the same order as those of NpHR, HsHR, and MrHR (2–6 mM) (10, 11, 18, 33, 42), but was approximately 100-fold lower than that of SyHR (approximately 0.1 mM) (25) and approximately 3- and 5-fold larger than those of NM-R3 (24 mM) and *Fulvimarina pelagi* HTCC 2506^T^ rhodopsin (FR) (84 mM), respectively (16, 35). In addition, the acid dissociation constant, p*K*_a_, of the Schiff base was dependent on the Cl^−^ concentration (Fig. S2D), which is similar to archaeal HsHR and NpHR (39). At low concentrations of NaCl (0 - several tens of mM), the absorption band of the deprotonated Schiff base appeared in the shorter wavelength region at approximately 380 nm (Fig. S2B). Therefore, subsequent experiments were performed in the presence of more than 100 mM NaCl.

The Cl^−^-transporting photoreaction, called a photocycle, of RmHR was analyzed using a time-resolved flash-photolysis technique. Fig. S3A shows the flash-induced light-minusdark difference absorption spectra in the 10-μs to 388-ms time domain. After flash excitation, photointermediates with absorption bands at approximately 460 nm and 610 nm, respectively, were generated with the concomitant disappearance of the initial state absorption band at approximately 550 nm. Time-dependent absorption changes in these three absorption bands are shown in Fig. S3B. Data were simultaneously fit to the exponential decay function with 4 exponents, which was the same number as NpHR (10, 18), indicating that RmHR passed through 4 kinetically intermediate states, P_1_–P_4_, in its photocycle. The time constants between each transition, τ_1_–τ_4_, were 0.105, 0.483, 2.14, and 23.5 ms, respectively. According to the sequential model (10, 18), we calculated the absolute spectra of the P_1_–P_4_ states of RmHR in the presence of 1,000 mM NaCl, as shown in Fig. S3C. The spectrum of P_0_ corresponded to the initial state pure absorption spectrum of RmHR, which was extracted from the experimentally measured absorption spectrum (see the Materials and Methods section). The P_1_ and P_2_ states each contained a blue-shifted photointermediate with a similar absorption maximum at 510 nm. Analogous to NpHR, we tentatively assigned these intermediates as L_1_- and L_2_-like, respectively. The following P_3_ state contained at least two photointermediates with absorption maxima at 520 and 590 nm, which we tentatively assigned as N- and O-like intermediates, respectively, by referring to the P_3_ state of NpHR. In the case of NpHR, the molar ratio of N and O changed in a Cl^−^ concentration-dependent manner (10, 18). In order to evaluate this phenomenon, we performed flash photolysis measurements on RmHR with varying Cl^−^ concentrations. It is important to note that the Cl^−^ concentration-dependent absorption change was significant in the P_3_ state (Fig. S4). Fig. S3D shows the Cl^−^-dependent change in the molar ratio of N-like and O-like intermediates between 100 mM and 4 M NaCl. The fraction of the N-like intermediate increased with elevations in the Cl^−^ concentration, indicating that this intermediate is the Cl^−^-binding form. In contrast, the fraction of the O-like intermediate decreased with the increase in the Cl^−^ concentration, indicating that the O-like intermediate is the Cl^−^-releasing form. Data were simultaneously fit by the skewed Gaussian function in order to identify the fractions of the N-like and O-like intermediates. As shown in Fig. S3E, we plotted these fractions against NaCl concentrations and then estimated the dissociation constant for Cl^−^ release, *K*_d,rel_, as 308±45.6 mM using the Hill equation. The value was approximately 40-fold larger than that of the initial state (*K*_d,int._=7.6 [mM]), the behavior of which was the same as NpHR, whereas the magnitude of the change was different (*K*_d,int._=2 [mM] and *K*_d,rel_=1.2 [M]) (33). In the P_4_ state (Fig. S3C), a photointermediate with the same absorption maximum at 540 nm as the initial state (P_0_) was identified, and we named it the RmHR′-intermediate by referring to other anion-pumping rhodopsins, including NpHR (18, 35). Analogous to NpHR, in the O-like-to-RmHR’ transition, Cl^−^ was entered into the initial binding site on the extracellular side of RmHR, which was supported by the blue-shifted absorption from O-like to RmHR’ and the same absorption maximum of RmHR’ as that of the initial state. We summarized the Cl^−^ transporting photocycle model of RmHR in Fig. S3F. The initially captured Cl^−^ on the extracellular side of RmHR was transferred to the intracellular side in the L_2_-like-to-N-like transition and was then released into the intracellular bulk space in the N-like-to-O-like transition. The next Cl^−^ was entered into the initial binding site through the intracellular side of the molecule in the O-like to RmHR’ and RmHR’ to initial state transitions. The mechanism was similar to that of NpHR (18), but significantly different from that of the bacterial anion pumps, NM-R3 and FR, which are evolutionally distant from RmHR (Table 2) (Fig. 2) (16, 35).

### Phylogenetic analyses based on retinal biosynthesis pathway genes

**Fig. 4:**
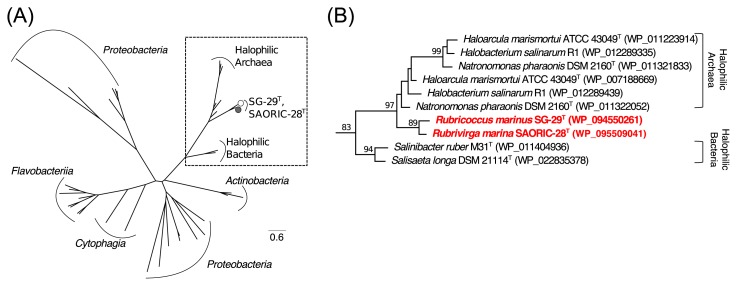
Unrooted maximum-likelihood phylogenetic tree of the *blh* gene. Amino acid sequences were aligned using CLUSTALW, and evolutionary distances were estimated using the LG with Freg model. The tree was constructed using bootstrap values based on 1,000 replications; evolutionary analyses were conducted in MEGA 6.0. (A) Open and closed gray circles indicate the positions of strains SG-29^T^ and SAORIC-28^T^, respectively. (B) Detailed phylogenetic relationship of the *blh* gene among *Flavobacteriia*, *Cytophagia*, halophiles, strain SG-29^T^, and strain SAORIC-28^T^. Bootstrap values >80% are indicated as a percentage of the replicates tested.

Functional and spectroscopic analyses revealed that RmHR is a light-driven inward Cl^−^-pumping rhodopsin similar to the HR of halophilic archaea rather than to ClR (*e.g.*, NM-R3 and FR) of marine flavobacteria. In order to further examine the evolutionary history of RmHR, we performed phylogenetic analyses on retinal biosynthesis pathway genes, such as *crtY* (lycopene cyclase) and *blh* (15,15′-β-carotene dioxygenase). The enzymes encoded by the *crtY* and *blh* genes catalyze the terminal cyclization reaction from all-*trans*-lycopene to all *trans*-β-carotene and cleave β-carotene to produce two molecules of all-*trans*-retinal as a chromophore of rhodopsin. ML phylogenetic trees based on the *blh* and *crtY* genes are shown in Fig. 4 and S5. Regarding SAORIC-476^T^, since this strain lacks rhodopsin and *blh* genes, only the *crtY* gene was used in this analysis. In the phylogenetic tree of the *blh* gene, although each cluster was mainly formed according to the taxonomic group, the *blh* genes of SG-29^T^ and SAORIC-28^T^ in the class *Cytophagia* belonged to the lineage containing halophilic archaea and bacteria (Fig. 4). The phylogenetic tree based on *crtY* showed that three clusters: *Bacteroidetes crtY* and *Proteobacteria crtY* and *crtYcd*, had formed, and the genes of strains SG-29^T^, SAORIC-28^T^, and SAORIC-476^T^ were included in the *crtYcd* cluster (Fig. S5). Carotenoid cyclases such as *crtY* are considered to be diversified by LGT or gene duplication, and some *Actinobacteria* (20), archaea (12), and *Bacteroidetes* (19) contain *crtYcd*. Although the similarity between the *crtYcd* and *crtY* genes is not high, the *crtYcd* gene is in a carotenoid biosynthesis gene cluster and has been shown to encode an enzyme that functions as a lycopene cyclase-like *crtY* gene. Halophilic archaea, the genera *Natronomonas*, *Halobacterium*, and *Haloarcula*, and halophilic bacteria, *S. ruber* M31^T^ and *S. longa* DSM 21114^T^, possess the *crtYcd*, but not *crtY* gene. Similar to the phylogenetic tree of the *blh* gene, the *crtYcd* genes of *Rubricoccus* and *Rubrivirga* were not close to the genes of the *Bacteroidetes crtY* clade, whereas these *crtYcd* genes were closely related to the halophilic archaeal ones. Although the family *Rhodothermaceae* belongs to the class *Cytophagia* in the phylum *Bacteroidetes*, *blh* and lycopene cyclase genes formed a phylogenetic lineage that is distinct from these genes in *Bacteroidetes*.

Previous genomic and phylogenetic analyses showed that *S. ruber* M31^T^ acquired several genes from halophilic archaea via LGT (23). Gene transfer beyond a domain may have occurred because *Salinibacter* and halophilic archaea dominate in the same niche, such as a hypersaline environment. The best example of this gene transfer is the HR gene shared by *Salinibacter* and halophilic archaea. In addition, not only this case, but also other genes (*e.g.*, *blh*, *crtYcd*, the cytochrome c oxidase gene, and nitrous oxide reductase gene) were shared among halophilic bacteria and archaea. Our results based on the phylogenetic analysis suggest that the rhodopsin gene and its related genes were shared not only between *Salinibacter* and halophilic archaea, but also among those halophiles and marine bacteria (*R. marinus* SG-29^T^ and *R. marina* SAORIC-28^T^). Furthermore, these results indicate that the common ancestor of the genera *Rubricoccus* and *Rubrivirga* acquired rhodopsin and its related genes from halophilic archaea via LGT in a hypersaline environment, as reported previously (23).

### Visualizing genomic flanking regions of rhodopsin and *blh* genes

The results showing that several genes were shared among the halophiles, *Rubricoccus* and *Rubrivirga*, indicated that a common ancestor of strains SG-29^T^, SAORIC-28^T^, and SAORIC-476^T^ inhabited the same environment, such as salt lakes, as halophiles. In order to clarify whether other genes were acquired together with the rhodopsin and *blh* genes, we compared the genomic flanking regions of these genes among the genera *Rubricoccus* and *Rubrivirga* and the strains possessing genes closely related to this rhodopsin or the *blh* gene (Fig. S6). The results of the analysis showed that there was low similarity around rhodopsin genes (Fig. S6A). This dissimilarity may suggest that only Cl^−^-pumping rhodopsin genes were acquired by LGT or that a gene transfer event has not recently occurred. The same analysis based on the *blh* gene also showed no remarkable similarity to the flanking regions of the rhodopsin gene. The flanking regions of *R. profundi* SAORIC-476^T^ lacking the rhodopsin and *blh* genes showed the presence of multiple conserved genes between SAORIC-28^T^ and SAORIC-476^T^ (Fig. S6B). This result suggested that only the *blh* gene was deleted in SAORIC-476^T^ or acquired in SAORIC-28^T^. The results of a BLAST analysis revealed that although the numbers of potential LGT of strains SG-29^T^, SAORIC-28^T^, and SAORIC-476^T^ were lower than that of *S. ruber* M31^T^, these numbers were approximately 2- to 4-fold higher than other phylum *Bacteroidetes* strains (Fig. S7). This result suggests that genes in strains SG-29^T^, SAORIC-28^T^, and SAORIC-476^T^ may be shared with *H. salinarum*, similar to *S. ruber*.

In conclusion, we herein report a novel light-driven inward Cl^−^-pumping rhodopsin (RmHR), similar to the HR of halophilic archaea, in marine isolates of the family *Rhodothermaceae* in the phylum *Bacteroidetes*. Spectroscopic assays also showed that the properties of RmHR resembled those of HR from the aspect of the intermediate states and mechanisms of Cl^−^ uptake and release. Phylogenetic analyses revealed that strains of the genera *Rubricoccus* and *Rubrivirga* may have acquired some genes by LGT, including the rhodopsin and *blh* genes from halophilic archaea. These results suggest that these genera and halophilic archaea had once lived in the same environment. Collectively, the present results provide insights into gene sharing between marine bacteria and halophilic archaea and facilitate our understanding of the evolutionary processes occurring in the ecologically diverse environments of this family.

## Supplementary Material



## Figures and Tables

**Table 1:** Comparison of important residues, particularly motifs, among ion pumps.

Residue numbers in BR (above) and PR (below)														

			85	86	87	88	89	90	91	92	93	94	95	96
Rhodopsin type	Taxonomic group	Motif	97	98	99	100	101	102	103	104	105	106	107	108
**BR**	*Halobacterium salinarum* R1	DTD	D	W	L	F	T	L	P	L	L	L	L	D
**PR**	*Ca.* Pelagibacter ubique HTCC 1062	DTE	D	W	L	I	T	V	P	L	L	M	L	E
**ClR**	*Nonlabens marinus* S1-08^T^	NTQ	N	W	M	A	T	I	P	C	L	L	L	Q
**SyHR**	*Synechocystis* sp. PCC 7506	TSD	T	W	F	L	S	T	P	L	L	L	L	D
**HR**	*Salinibacter ruber* M31^T^	TSA	T	W	A	F	S	T	P	F	I	L	L	A
**HR**	*Halobacterium salinarum* R1	TSA	T	W	A	L	S	T	P	M	I	L	L	A
**RmXeR**	*Rubricoccus marinus* SG-29^T^	DTA	D	W	V	V	T	T	P	L	L	L	T	A
**R28XeR**	*Rubrivirga marina* SAORIC-28^T^	DTA	D	W	V	V	T	T	P	L	L	L	A	A
**RmHR**	*Rubricoccus marinus* SG-29^T^	TSA	T	W	F	T	S	T	P	L	L	L	L	A
**R28HR1**	*Rubrivirga marina* SAORIC-28^T^	TSA	T	W	F	L	S	T	P	L	L	L	L	A
**R28HR2**	*Rubrivirga marina* SAORIC-28^T^	TTD	T	W	F	L	T	T	P	L	L	L	L	D

**Table 2:** Comparison of spectroscopic properties of RmHR with archaeal and cyanobacterial HRs and marine bacterial ClRs.

Rhodopsin (Type)	Retinal isomer (dark/light) (%)	*K*_d,int_/*K*_d,rel_ (mM)	Schiff base p*K*^a^	Photo-intermediates	Existence of N-like–O-like equilibrium	Similarity of the photocycle scheme	References
RmHR (marine bacterial HR)	99.6/96.2	7.6/308	7.3 (0 M NaCl)–10 (4 M NaCl)	(K), L_1_, L_2_, N, O, RmHR’	Yes	(This study)	This study
HsHR (archaeal HR)	50/86	2.6/N.D.	Approx. 8 (10 mM NaCl)–Approx. 11.5 (below 1 M NaCl)	K, L_1_, L_2_, N	No	Different from RmHR	38, 39, 42
NpHR (archaeal HR)	83/77	2/1200	Approx. 8 (10 mM NaCl)–Approx. 10 (below 1 M NaCl)	(K), L_1_, L_2_, N, O, NpHR’	Yes	Similar to RmHR	10, 18, 33, 37, 39
NM-R3 (marine bacterial ClR)	90.3/91.6	24/N.D.	N.D.	K, L, N, O_1_, O_2_, NM-R3’	No	Different from RmHR	35
FR (marine bacterial ClR)	96.4/86.6	84/N.D.	N.D.	K, L, O, FR’	N.D.	Different from RmHR	16
MrHR (cyanobacterial HR)	>98/>98	1.99/N.D.	N.D.	(K), L, N, O, MrHR’	Yes	Different from RmHR	9, 11
SyHR (cyanobacterial HR)	N.D./97.5	0.1/N.D.	10.6 (1 M NaCl)	K, L, N, O, SyHR’	Yes	Similar to RmHR	25

N.D. indicates “Not determined”.
