# Supplementary material for: Presence of a Haloarchaeal Halorhodopsin-Like Cl− Pump in Marine Bacteria
Source: Microbes Environ. 2018 Mar 29;33(1):89–97. doi: 10.1264/jsme2.ME17197 (PMC5877348; doi:10.1264/jsme2.ME17197)
Supplement: Supplementary file 1 [file 33_89_s1.pdf]

Table S1. General genomic characteristics of *Rubricoccus marinus* SG-29$^T$, *Rubrivirga marina* SAORIC-28$^T$ and *Rubrivirga profundi* SAORIC-476$^T$.

| Name | Isolation source | Genome size (Mbp) | Coverage | G+C content (%mol) | CDS number | $N_{50}$ (bp) | Scaffold | Rhodopsin gene | Reference |
|---|---|---|---|---|---|---|---|---|---|
| *Rubricoccus marinus* SG-29$^T$ | Sea water (50 m) | 4.43 | 58 | 69.0 | 3847 | 167,811 | 15 | + | (24) |
| *Rubrivirga marina* SAORIC-28$^T$ | Deep sea water (3000 m) | 4.98 | 81 | 72.5 | 4267 | 326,174 | 11 | + | This study |
| *Rubrivirga profundi* SAORIC-476$^T$ | Deep sea water (3000 m) | 4.48 | 86 | 71.3 | 3842 | 447,061 | 19 | – | This study |

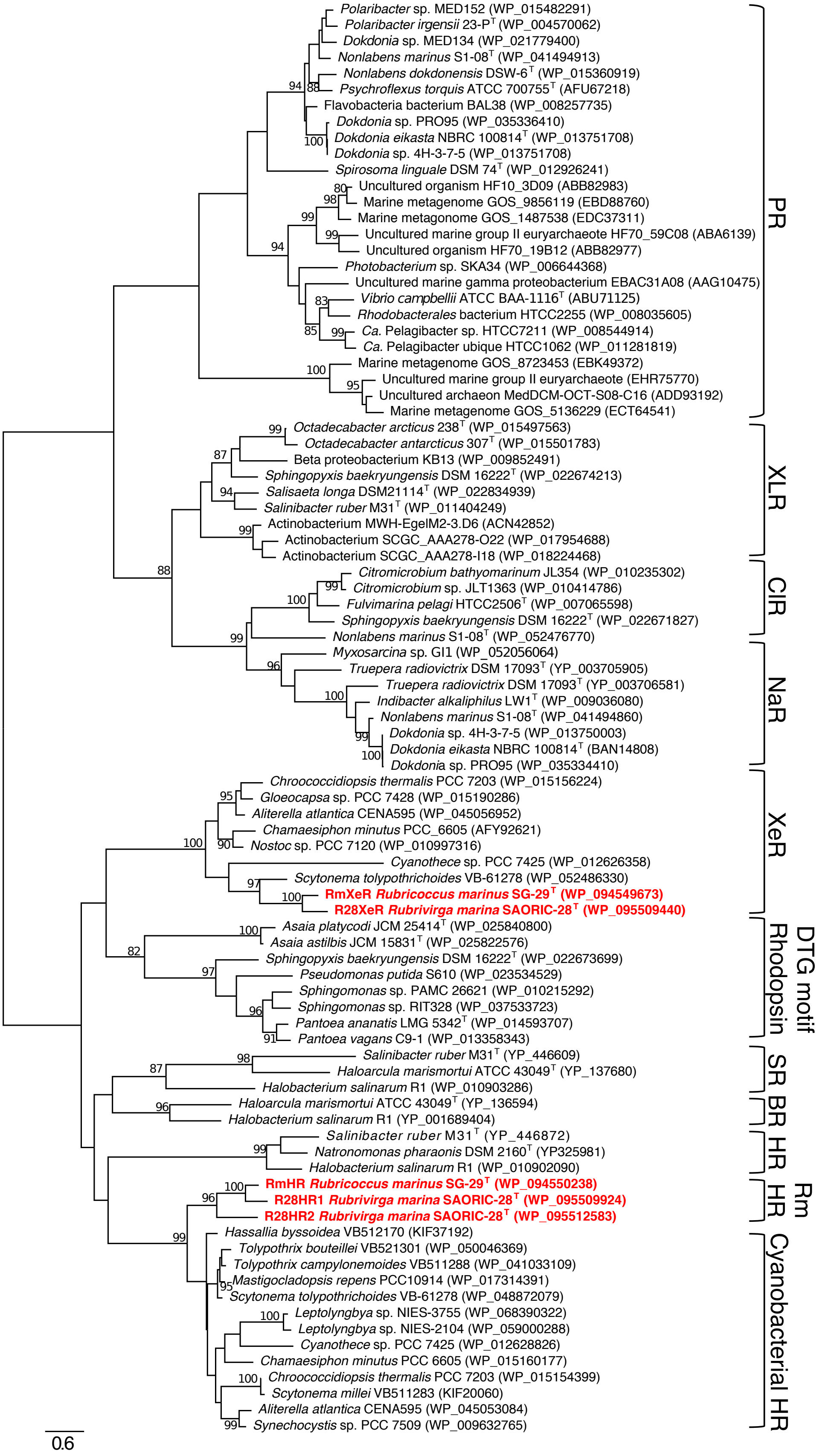

Polaribacter sp. MED152 (WP_015482291)
Polaribacter irgensii 23-P$^{T}$ (WP_004570062)
Dokdonia sp. MED134 (WP_021779400)
Nonlabens marinus S1-08$^{T}$ (WP_041494913)
Nonlabens dokdonensis DSW-6$^{T}$ (WP_015360919)
Psychroflexus torquis ATCC 700755$^{T}$ (AFU67218)
Flavobacteria bacterium BAL38 (WP_008257735)
Dokdonia sp. PRO95 (WP_035336410)
Dokdonia eikasta NBRC 100814$^{T}$ (WP_013751708)
Dokdonia sp. 4H-3-7-5 (WP_013751708)
Spirosoma linguale DSM 74$^{T}$ (WP_012926241)
Uncultured organism HF10_3D09 (ABB82983)
Marine metagenome GOS_9856119 (EBD88760)
Marine metagonome GOS_1487538 (EDC37311)
Uncultured marine group II euryarchaeote HF70_59C08 (ABA6139)
Uncultured organism HF70_19B12 (ABB82977)
Photobacterium sp. SKA34 (WP_006644368)
Uncultured marine gamma proteobacterium EBAC31A08 (AAG10475)
Vibrio campbellii ATCC BAA-1116$^{T}$ (ABU71125)
Rhodobacterales bacterium HTCC2255 (WP_008035605)
Ca. Pelagibacter sp. HTCC7211 (WP_008544914)
Ca. Pelagibacter ubique HTCC1062 (WP_011281819)
Marine metagenome GOS_8723453 (EBK49372)
Uncultured marine group II euryarchaeote (EHR75770)
Uncultured archaeon MedDCM-OCT-S08-C16 (ADD93192)
Marine metagenome GOS_5136229 (ECT64541)
Octadecabacter arcticus 238$^{T}$ (WP_015497563)
Octadecabacter antarcticus 307$^{T}$ (WP_015501783)
Beta proteobacterium KB13 (WP_009852491)
Sphingopyxis baekryungensis DSM 16222$^{T}$ (WP_022674213)
Salisaeta longa DSM21114$^{T}$ (WP_022834939)
Salinibacter ruber M31$^{T}$ (WP_011404249)
Actinobacterium MWH-EgelM2-3.D6 (ACN42852)
Actinobacterium SCGC_AAA278-O22 (WP_017954688)
Actinobacterium SCGC_AAA278-I18 (WP_018224468)
Citromicrobium bathyomarinum JL354 (WP_010235302)
Citromicrobium sp. JLT1363 (WP_010414786)
Fulvimarina pelagi HTCC2506$^{T}$ (WP_007065598)
Sphingopyxis baekryungensis DSM 16222$^{T}$ (WP_022671827)
Nonlabens marinus S1-08$^{T}$ (WP_052476770)
Myxosarcina sp. GI1 (WP_052056064)
Truepera radiovictrix DSM 17093$^{T}$ (YP_003705905)
Truepera radiovictrix DSM 17093$^{T}$ (YP_003706581)
Indibacter alkaliphilus LW1$^{T}$ (WP_009036080)
Nonlabens marinus S1-08$^{T}$ (WP_041494860)
Dokdonia sp. 4H-3-7-5 (WP_013750003)
Dokdonia eikasta NBRC 100814$^{T}$ (BAN14808)
Dokdonia sp. PRO95 (WP_035334410)
Chroococcidiopsis thermalis PCC 7203 (WP_015156224)
Gloeocapsa sp. PCC 7428 (WP_015190286)
Aliterella atlantica CENA595 (WP_045056952)
Chamaesiphon minutus PCC_6605 (AFY92621)
Nostoc sp. PCC 7120 (WP_010997316)
Cyanothece sp. PCC 7425 (WP_012626358)
Scytonema tolypothrichoides VB-61278 (WP_052486330)
RmXeR Rubricoccus marinus SG-29$^{T}$ (WP_094549673)
R28XeR Rubrivirga marina SAORIC-28$^{T}$ (WP_095509440)
Asaia platycodi JCM 25414$^{T}$ (WP_025840800)
Asaia astilbis JCM 15831$^{T}$ (WP_025822576)
Sphingopyxis baekryungensis DSM 16222$^{T}$ (WP_022673699)
Pseudomonas putida S610 (WP_023534529)
Sphingomonas sp. PAMC 26621 (WP_010215292)
Sphingomonas sp. RIT328 (WP_037533723)
Pantoea ananatis LMG 5342$^{T}$ (WP_014593707)
Pantoea vagans C9-1 (WP_013358343)
Salinibacter ruber M31$^{T}$ (YP_446609)
Haloarcula marismortui ATCC 43049$^{T}$ (YP_137680)
Halobacterium salinarum R1 (WP_010903286)
Haloarcula marismortui ATCC 43049$^{T}$ (YP_136594)
Halobacterium salinarum R1 (YP_001689404)
Salinibacter ruber M31$^{T}$ (YP_446872)
Natronomonas pharaonis DSM 2160$^{T}$ (YP325981)
Halobacterium salinarum R1 (WP_010902090)
RmHR Rubricoccus marinus SG-29$^{T}$ (WP_094550238)
R28HR1 Rubrivirga marina SAORIC-28$^{T}$ (WP_095509924)
R28HR2 Rubrivirga marina SAORIC-28$^{T}$ (WP_095512583)
Hassallia byssoidea VB512170 (KIF37192)
Tolypothrix bouteillei VB521301 (WP_050046369)
Tolypothrix campylonemoides VB511288 (WP_041033109)
Mastigocladopsis repens PCC10914 (WP_017314391)
Scytonema tolypothrichoides VB-61278 (WP_048872079)
Leptolyngbya sp. NIES-3755 (WP_068390322)
Leptolyngbya sp. NIES-2104 (WP_059000288)
Cyanothece sp. PCC 7425 (WP_012628826)
Chamaesiphon minutus PCC 6605 (WP_015160177)
Chroococcidiopsis thermalis PCC 7203 (WP_015154399)
Scytonema millei VB511283 (KIF20060)
Aliterella atlantica CENA595 (WP_045053084)
Synechocystis sp. PCC 7509 (WP_009632765)
PR
XLR
ClR
NaR
XeR
DTG motif Rhodopsin
SR
BR
HR
Rm HR
Cyanobacterial HR
0.6

**Fig. S1. Phylogenetic tree of microbial rhodopsins.** Unrooted maximum-likelihood tree of microbial rhodopsin amino acid sequences. Amino acid sequences of microbial rhodopsins were aligned using CLUSTALW, and evolutionary distances were estimated using the LG with the Freg model. The tree was constructed using bootstrap values based on 1000 replications; evolutionary analyses were conducted in MEGA 6.0. Bootstrap values >80% are indicated as a percentage of replicates determined.

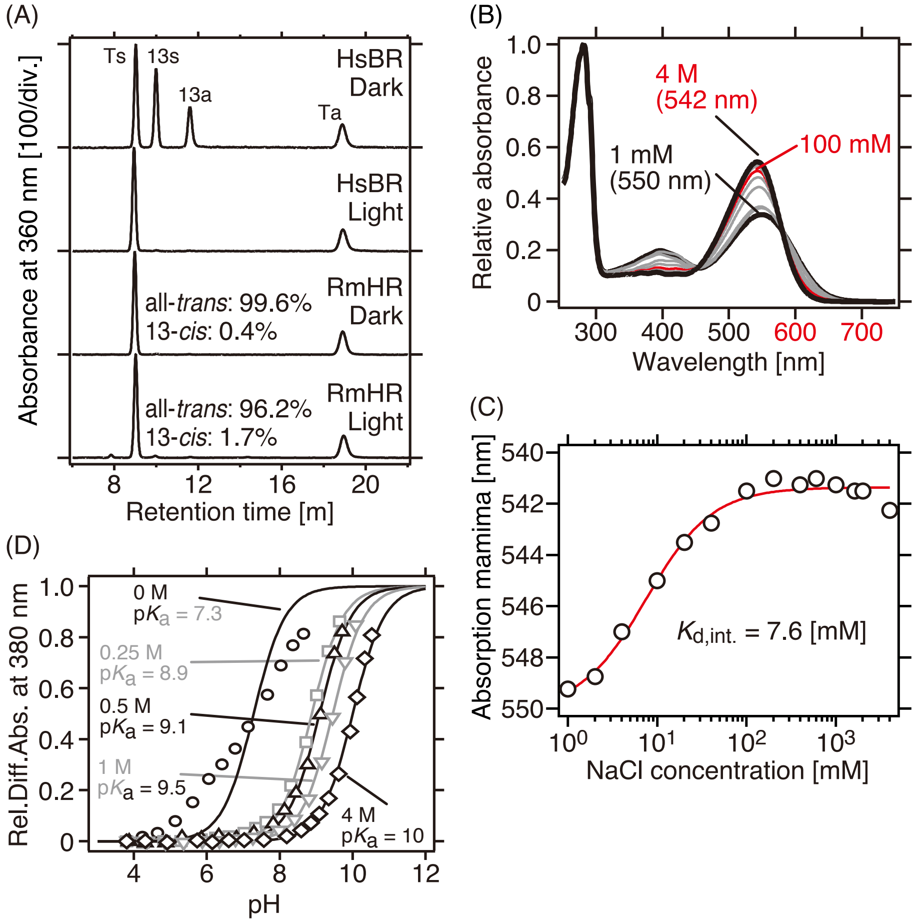


**Fig. S2. Photochemical properties of initial state RmHR.** (A) HPLC chromatograms of retinal oximes extracted from RmHR under dark and light conditions in the presence of 1 M NaCl. HsBR was used as a reference. Ts, Ta, 13s, and 13a represent the retinal configuration; all-*trans*-15-*syn*, all-*trans*-15-*anti*, 13-*cis*-15-*syn*, and 13-*cis*-15-*anti*, respectively. (B) UV-Vis absorption spectra of RmHR in the presence of 1 mM to 4 M NaCl. The spectrum at 100 mM NaCl is highlighted, where the absorption band corresponding to the deprotonated Schiff base (380 nm) disappeared. (C) The Hill plot for absorption maxima against the logarithm of the NaCl concentration. The dissociation

constant for $Cl^-$ in the initial state was estimated as 7.6 ± 1.7 mM. The fitting curve is represented by a red solid line. The fitting parameters were $a$ = 550 ± 0.79, $b$ = -8.7 ± 0.86, $n$ = 1.2 ± 0.23. (D) pH-dependent absorption changes at 380 nm. The acid dissociation constant, $pK_a$, was indicated in the figure in each sample condition (0 M, circle; 0.25 M, square; 0.5 M, upward triangle; 1 M, downward triangle; 4 M, diamond). Fitting curves are represented by solid lines.

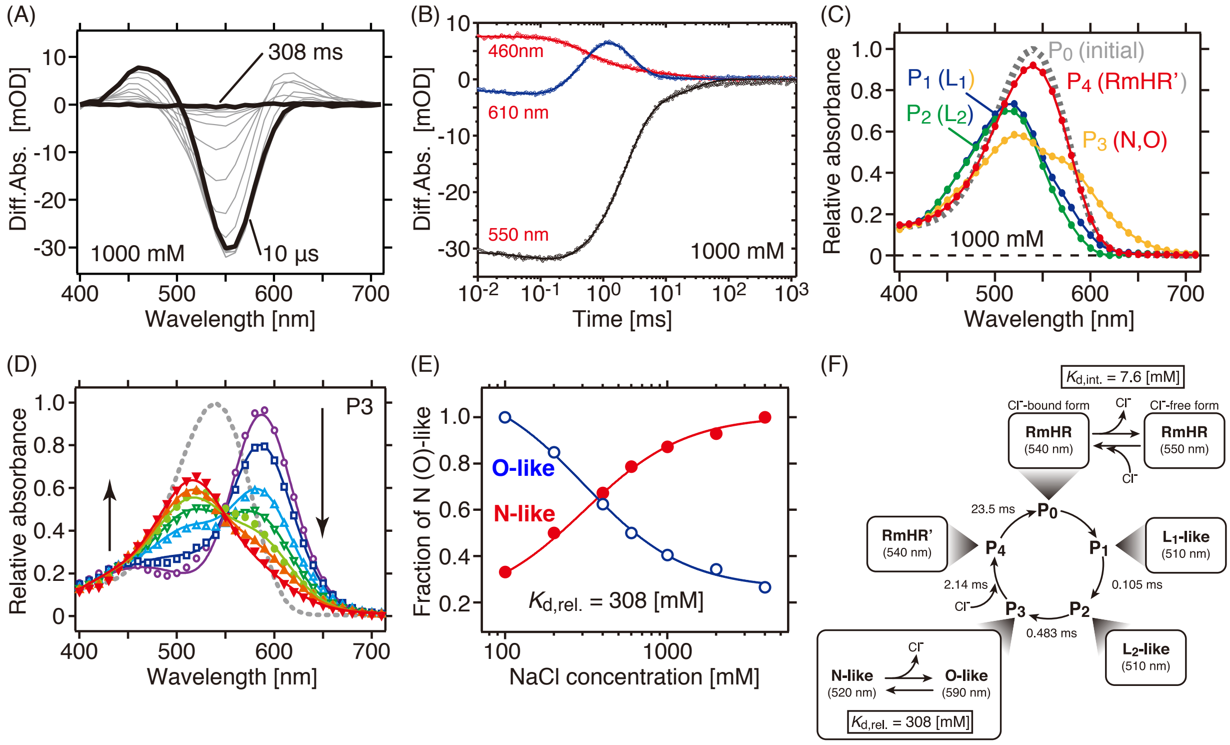


**Fig. S3. Photochemical properties of RmHR in its $Cl^-$-transporting photocycle.** (A) Flash-induced light-minus-dark absorption spectra in the 10 μs to 388 ms time domain in the presence of 1 M NaCl. The spectra of start and end times are highlighted. (B) Time-dependent absorption changes in RmHR in the presence of 1 M NaCl. Three wavelengths–460 nm, 550 nm, and 610 nm–are represented as red, black and blue circles, respectively. Fitting curves are represented by solid lines. (C) Absolute spectra of the kinetically defined photointermediate states $P_1$ – $P_4$ in the presence of 1 M NaCl are illustrated in blue, green, orange, and red closed-circles, respectively. The circles are connected by spline curves. Physically defined photointermediates are

described in parenthesis. $P_0$ denotes the initial state pure retinal absorption of the initial state RmHR illustrated in the gray broken line. (D) $Cl^-$-dependent absorption changes in the N-like (520 nm) and O-like (590 nm) ratio from 100 mM to 4 M (100 mM, purple circle; 200 mM blue square; 400 mM, light-blue upward triangle; 600 mM, green downward triangle, 1 M, light-green closed-circle; 2 M, orange upward triangle; 4 M, red downward triangle). The arrows indicate the direction of change with increasing NaCl concentrations. Fitting curves are represented by solid lines. The gray broken line denotes the initial state spectrum, $P_0$. The fitting parameters of the skewed Gaussian functions were $\lambda_{max}$ = 518 [nm], $A_{max}$ (maximum absorption) = 0.249, ρ (skewness of the absorption band) = 1.16, and Δν (half-bandwidths) = 3455 [$cm^{-1}$] for the main band of N-like intermediate, and $\lambda_{max}$ = 588 [nm], $A_{max}$ = 0.403, ρ = 1.06, and Δν = 4789 [$cm^{-1}$] for the main band of the O-like intermediate, respectively. (E) $Cl^-$-dependent changes in the fractions of N-like (red closed circle) and O-like (blue circle) photointermediates. The dissociation constant for $Cl^-$ release in the photocycle was estimated as 308 ± 27.9 mM. Fitting curves are indicated by solid lines, and the fitting parameters for both N-like and O-like fractions were $a$ = 0.188 ± 0.0491, $b$ = 0.819 ± 0.0606, $n$ = 1.40 ± 0.145, and $a$ = 1.16 ± 0.0535, $b$ = -0.899 ± 0.0659, $n$ = 1.40 ± 0.145, respectively. (F) The $Cl^-$-pumping photocycle model of RmHR.

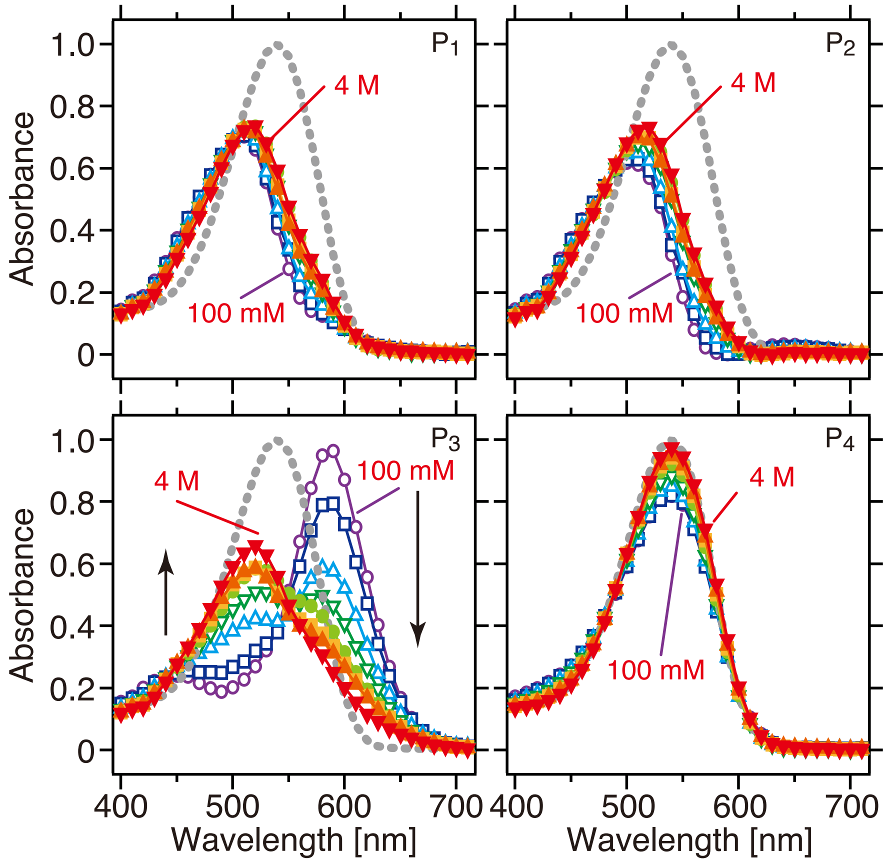


**Fig. S4. Absolute spectra of the kinetically defined photointermediate states $P_1$ – $P_4$ in the presence of 100 mM to 4 M NaCl (100 mM, purple circle; 200 mM blue square; 400 mM, light-blue upward triangle; 600 mM, green downward triangle, 1 M, light-green closed-circle; 2 M, orange upward triangle; 4 M, red downward triangle).** The circles were connected by spline curves. The gray broken lines correspond to the initial state pure retinal absorption of the initial state RmHR ($P_0$). The arrows indicate the direction of change with increasing NaCl concentrations.

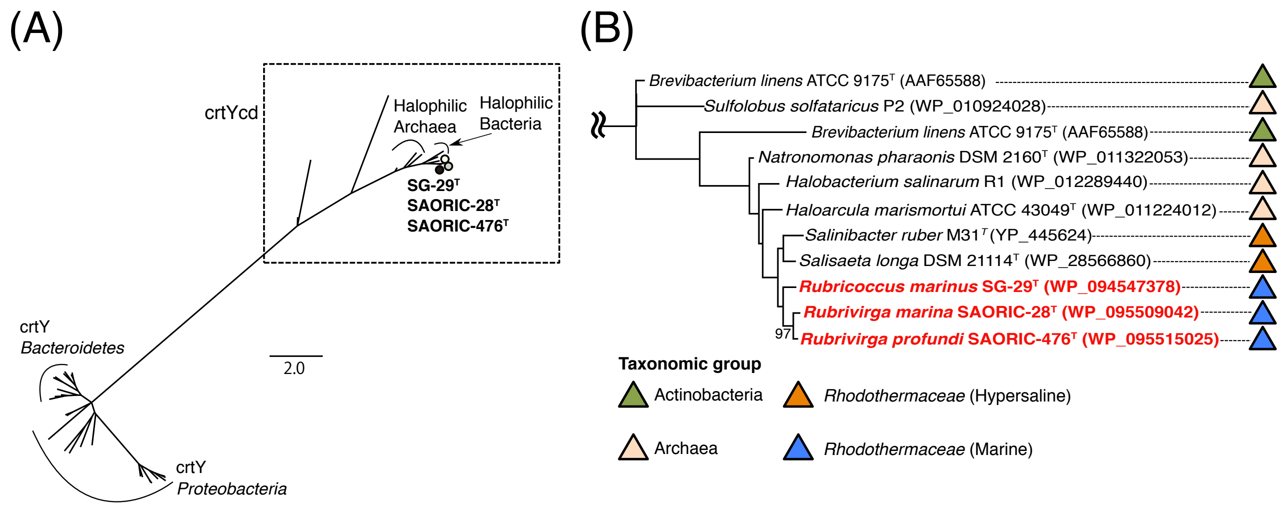


**Fig. S5. Unrooted maximum-likelihood phylogenetic tree of lycopene cyclase gene.** Amino acid sequences were aligned using CLUSTALW, and evolutionary distances were estimated using the LG with Freg model. The tree was constructed using bootstrap values based on 1000 replications; evolutionary analyses were conducted in MEGA 6.0. (A) Open circles and closed gray and black circles indicate the positions of strains SG-29$^T$, SAORIC-28$^T$ and SAORIC-476$^T$, respectively. (B) Detailed phylogenetic relationship of *crtYcd* gene. Bootstrap values >80% are indicated as a percentage of the replicates determined. The taxonomic groups for each strain are indicated by different colors of closed triangles.

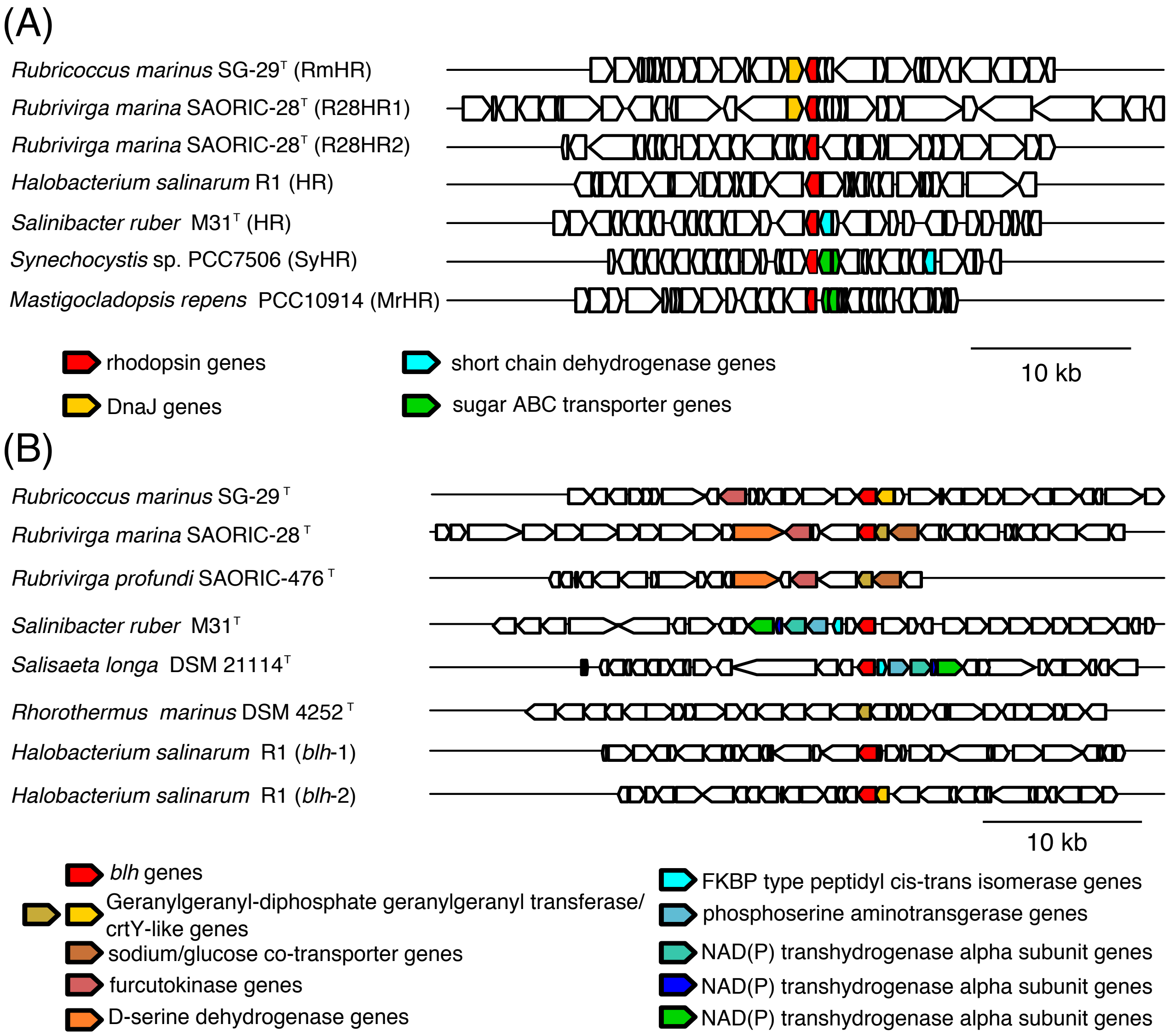


**Fig. S6. Genomic flanking regions of chloride ion pumping rhodopsin (A) and *blh* (B) genes.**

The positions of each rhodopsin and *blh* genes are indicated in red. Each orthologous gene is indicated in same color.

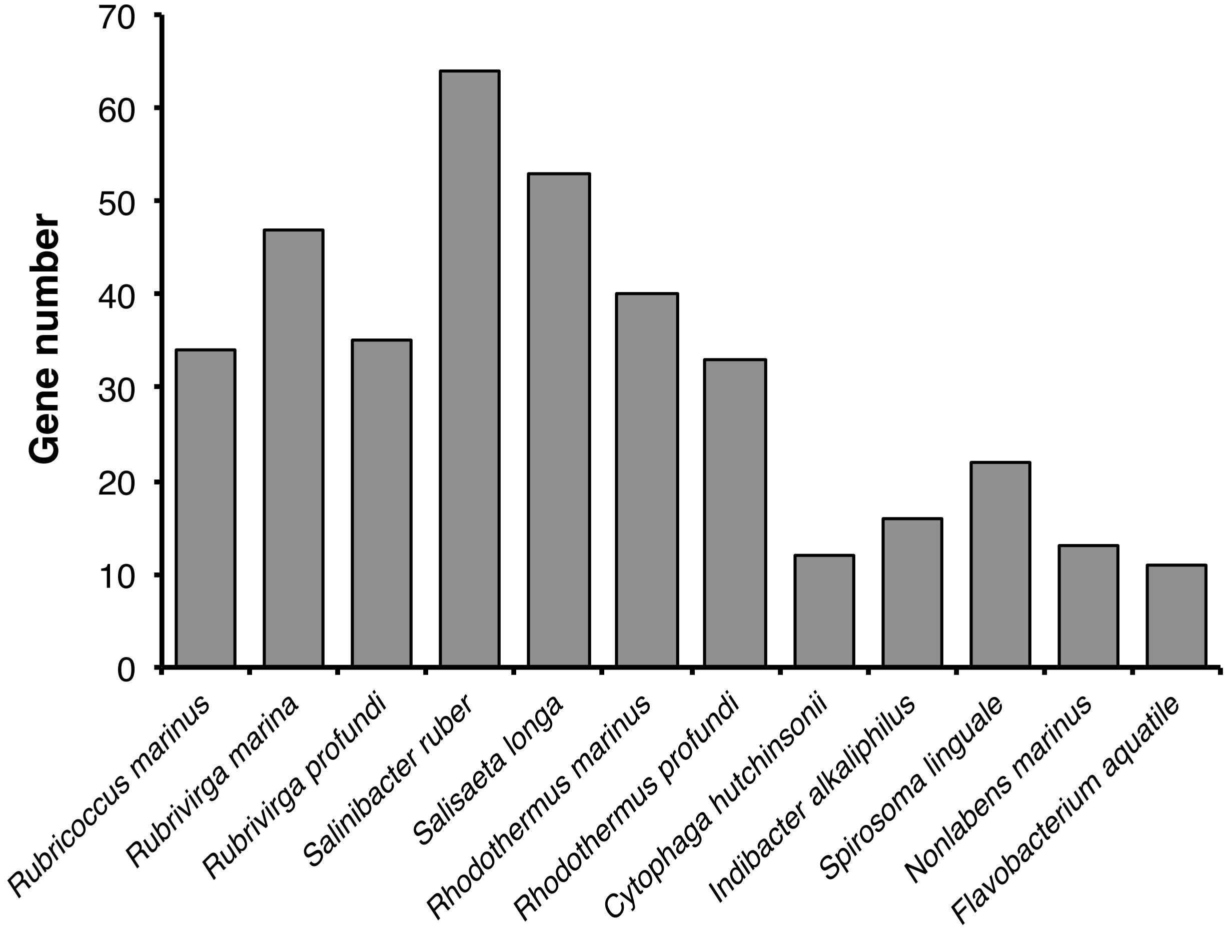


**Fig. S7. The number of potential lateral gene transfers from *H. salinarum* R1.** Each bar are represented the number of genes for which passed the threshold with BLAST hit to the genome of *H. salinarum* R1.
